# Supplementary material for: A dual function of the IDA peptide in regulating cell separation and modulating plant immunity at the molecular level
Source: eLife. 2024 Jun 18;12:RP87912. doi: 10.7554/eLife.87912 (PMC11186634; doi:10.7554/eLife.87912)
Supplement: Supplementary file 1. [file elife-87912-supp1.docx]

**Supplementary File 1: Peptide sequences**

| Peptide | Amino acid sequence |
| --- | --- |
| mIDA | PIPPSA**o**SKRHN |
| flg22 | QRLSTGSRINSAKDDAAGLQIA |
| IDA^ΔN69^ | PIPPSA**o**SKRH |
| PIP1 | RFVKHSG**o**SPSGPGH |

(**o** = hydroxyproline)
