# Supplementary material for: A dual function of the IDA peptide in regulating cell separation and modulating plant immunity at the molecular level
Source: eLife. 2024 Jun 18;12:RP87912. doi: 10.7554/eLife.87912 (PMC11186634; doi:10.7554/eLife.87912)
Supplement: Supplementary file 2. [file elife-87912-supp2.docx]

**Supplementary File 2: Primers sequences and function**

| Function | Primer name | Primer sequence 5’-3’ |
| --- | --- | --- |
| Cloning promoter FLS2 | PromFLS2 -988bp Attb1 | 5’GGGGACAAGTTTGTACAAAAAAGCAGGCTTA GAAGTTGTGAATTGTGAT’3 |
| Cloning promoter FLS2 | PromFLS2 -988bp Attb2 | 5’GGGGACCACTTTGTACAAGAAAGCTGGGTA GGTTTAGACTTTAGAAGA’3 |
| Genotype hsl2 | Hsl2 LP | 5’CGTCTTGAGCTAGCCAACAAC’3 |
| Genotype hsl2 | Hsl2 RP | 5’GTCCAATCAAGTGGAGAAACG’3 |
| Genotype hae | Haesa LP | 5’CACCTTCCTTCTCTCCATTCC’3 |
| Genotype hae | Haesa RP | 5’GTTCGAGAAGTGACAAGCGAG’3 |
| Genotype SALK-lines | Lbb1- | 5’GCGTGGACCGCTTGCTGCAACT’3 |
| Genotype rbohd SALK_070610C | LP_SALK_070610C | 5’TTTCAACGCCTTTTGGTACAC’3 |
| Genotype rbohd SALK_070610C | RP_SALK_070610C | 5’GTTACCTATTCTTTTGCCGGG’3 |
| Genotype rbohf SALK_059888 | LP_SALK_059888 | 5’CAAAGAGCTCTTCGTGGTTTG’3 |
| Genotype rbohf SALK_059888 | RP_SALK_059888 | 5’TCTCTATTGTATCTTGTGTCACCG’3 |
| Genotype fls2 SALK_062054 | FLS2_SALK_062054 Fw | 5’GGTTCGATTCCTTCTGGAATC’3 |
| Genotype fls2 SALK_062054 | FLS2_SALK_062054 Rv | 5’CCTGAGTTTTTGAAGCTTCCC’3 |
| qPCR | 33.FRK1 Fw | 5’AACTTAGGAGACTATTTGGCAGGTAA’3 |
| qPCR | 33.FRK1 Rv | 5’TGCATCTAATGATATCTTCAACCTCT’3 |
| qPCR | 63.PEP3 Fw | 5’GCGAGGAAGATGAGAGTATCG’3 |
| qPCR | 63.PEP3 Rv | 5’TCAATGGTCATGCCATCTTCT’3 |
| qPCR | 91.MYB51 Fw | 5’GGCCAATTATCTTAGACCTGACA’3 |
| qPCR | 91.MYB51 Rv | 5’CCACGAGCTATAGCAGACCATT’3 |
| qPCR reference gene | act2int2 sense | 5’CCCTGAGGAGCACCCAGTTCTACTC’3 |
| qPCR reference gene | act2int2 antisense | 5’CCGCAAGATCAAGACGAAGGATAGC’3 |
