# Supplementary material for: A dual function of the IDA peptide in regulating cell separation and modulating plant immunity at the molecular level
Source: eLife. 2024 Jun 18;12:RP87912. doi: 10.7554/eLife.87912 (PMC11186634; doi:10.7554/eLife.87912)
Supplement: Supplementary file 3. — See Figure 2—figure supplement 1a for flower developmental stages (p2-p6). CNGC = CYCLIC NUCLEOTIDE GATED CHANNEL, RBOH = RESPIRATORY BURST OXIDASE PROTEIN, IDA = INFLORESCENCE DEFICIENT IN ABSCISSION, HSL2 = HAESA LIKE 2. [file elife-87912-supp3.docx]

**Supplementary File 3: Relative expression of genes of the CNGC and RBOH gene families during the onset of abscission** (data from (Cai & Lashbrook, 2008)). See Sup Fig. 5a for flower developmental stages (p2-p6). CNGC = CYCLIC NUCLEOTIDE GATED CHANNEL, RBOH = RESPIRATORY BURST OXIDASE PROTEIN, IDA = INFLORESCENCE DEFICIENT IN ABSCISSION, HSL2 = HAESA-LIKE 2.

| ***Locus ID*** | ***Name/Flower developmental stages*** | **p2** | **p3** | **p4** | **p5** | **p6** |
| --- | --- | --- | --- | --- | --- | --- |
| ***CNGCs*** |  |  |  |  |  |  |
| *At5g53130* | *CNGC1* | 291,9217 | 310,4919 | 495,7432 | 478,204 | 469,1732 |
| *At5g15410* | *CNGC2* | 1052,741 | 707,3286 | 626,9068 | 564,8327 | 557,5938 |
| *At5g54250* | *CNGC4* | 123,1472 | 193,9821 | 166,5376 | 176,2532 | 152,5896 |
| *At5g57940* | *CNGC5* | 691,9364 | 691,89 | 689,136 | 693,1147 | 396,6692 |
| *At2g23980* | *CNGC6* | 378,221 | 537,386 | 463,2181 | 502,9967 | 418,4082 |
| *At1g15990* | *CNGC7* | 42,53905 | 4,013366 | 3,253884 | 4,808204 | 7,830878 |
| *At1g19780* | *CNGC8* | 127,1805 | 63,14431 | 12,499 | 26,13849 | 11,25919 |
| *At4g30560* | *CNGC9* | 1115,91 | 673,8271 | 209,3169 | 197,1013 | 218,3005 |
| *At1g01340* | *CNGC10* | 92,29875 | 237,8649 | 115,0834 | 167,9021 | 116,888 |
| *At2g46440;At2g46430* | *CNGC11; CNGC3* | 3,751632 | 19,02687 | 101,7517 | 75,33781 | 97,46469 |
| *At2g46450* | *CNGC12* | 73,57635 | 154,3836 | 194,2215 | 256,2537 | 199,3865 |
| *At4g01010* | *CNGC13* | 26,81946 | 27,13074 | 36,37046 | 13,7691 | 35,73786 |
| *At2g24610* | *CNGC14* | 24,62137 | 24,55057 | 37,37278 | 32,75482 | 26,07159 |
| *At2g28260* | *CNGC15* | 26,53137 | 39,16623 | 21,37623 | 29,06457 | 22,87302 |
| *At3g48010* | *CNGC16* | 40,68595 | 12,40819 | 1,003874 | 1,432107 | 6,756874 |
| *At4g30360* | *CNGC17* | 127,9115 | 162,4681 | 216,6674 | 191,8812 | 157,5401 |
| *At5g14870* | *CNGC18* | 189,1577 | 98,09141 | 37,70282 | 29,79446 | 15,07226 |
| *At3g17690* | *CNGC19* | 4,532605 | 1,830825 | 5,04741 | 5,905303 | 3,699505 |
| *At3g17700* | *CNGC20* | 52,89588 | 94,00054 | 107,4842 | 78,12915 | 40,67315 |
|  |  |  |  |  |  |  |
| ***RBOHs*** |  |  |  |  |  |  |
| *At5g07390* | *RBOHA* | 4,185314 | 3,00695 | 3,438994 | 3,472965 | 3,41175 |
| *At1g09090* | *RBOHB* | 1,006999 | 1,055189 | 1,824025 | 1,175719 | 1,696683 |
| *At5g51060* | *RBOHC* | 20,71598 | 69,57144 | 13,97975 | 9,319057 | 8,847032 |
| *At5g60010* | *RBOHD* | 1150,998 | 603,4534 | 601,9696 | 805,7448 | 885,5323 |
| *At1g19230* | *RBOHE* | 7,372654 | 6,069166 | 4,934652 | 9,946411 | 6,019031 |
| *At1g64060* | *RBOHF* | 200,0895 | 222,463 | 297,3 | 449,7607 | 873,9841 |
| *At4g25090* | *RBOHG* | 4,048472 | 3,046103 | 3,217067 | 2,755258 | 5,142675 |
| *At5g60010* | *RBOHH* | 94,40216 | 65,08288 | 18,12167 | 26,1705 | 18,26213 |
| *At4g11230* | *RBOHI* | 11,76717 | 10,91102 | 17,55314 | 17,93307 | 11,61079 |
| *At3g45810* | *RBOHJ* | 99,10577 | 95,51017 | 71,29349 | 66,03646 | 63,07515 |
|  |  |  |  |  |  |  |
| ***IDA signaling pathway*** |  |  |  |  |  |  |
| *At1g68765* | *IDA* | 8,245425 | 6,850307 | 32,37852 | 224,6666 | 1268,797 |
| *At4g28490* | *HAESA* | 336,4576 | 811,4685 | 4950,168 | 6327,225 | 8082,581 |
| *At5g65710* | *HSL2* | 2517,483 | 4441,616 | 7633,162 | 8056,462 | 7993,356 |
